# An Emergent Change in Epidemiologic and Microbiological Characteristics of Bloodstream Infections in Adults With Febrile Neutropenia Resulting From Chemotherapy for Acute Leukemia and Lymphoma at Reference Centers in Chile, Ecuador, and Peru

**DOI:** 10.1093/ofid/ofae052

**Published:** 2024-02-01

**Authors:** Ricardo Rabagliati, Grace Salazar, Giancarlo Pérez-Lazo, Maria Paz Iturrieta, Diana Portillo, Carmen Soria-Segarra, María José Ojeda, Jimena Flores, Margarita Galarza, Roxana Sandoval-Ahumada, Pablo Cartes Aguilera, Lady Dimitrakis, Fabiola Avelga Reinoso, Patricia Garcia

**Affiliations:** Departamento de Enfermedades Infecciosas del Adulto, Escuela de Medicina, Pontificia Universidad Católica de Chile, Santiago, Chile; Hospital de Especialidades Eugenio Espejo, Quito, Ecuador; Hospital Nacional Guillermo Almenara Irigoyen, EsSalud, Lima, Perú; Hospital Sótero del Río, Santiago, Chile; Instituto Nacional de Enfermedades Neoplásicas, Lima, Perú; Universidad Católica Santiago de Guayaquil, Guayaquil, Ecuador; Departamento de Enfermedades Infecciosas del Adulto, Escuela de Medicina, Pontificia Universidad Católica de Chile, Santiago, Chile; Departamento de Enfermedades Infecciosas del Adulto, Escuela de Medicina, Pontificia Universidad Católica de Chile, Santiago, Chile; Laboratorio Microbiología, Hospital Eugenio Espejo, Quito, Ecuador; Hospital Nacional Guillermo Almenara Irigoyen, EsSalud, Lima, Perú; Laboratorio Microbiología, Hospital Sótero del Río, Santiago, Chile; Laboratorio Microbiología, Sociedad Lucha contra el Cáncer SOLCA, Guayaquil, Ecuador; Laboratorio Microbiología, Sociedad Lucha contra el Cáncer SOLCA, Guayaquil, Ecuador; Departamento de Laboratorios Clínicos, Escuela de Medicina, Pontificia Universidad Católica de Chile, Santiago, Chile

**Keywords:** bloodstream infection, febrile neutropenia, gram-negative rods, multidrug resistance

## Abstract

**Background:**

Febrile neutropenia is a life-threatening condition commonly observed in patients with hematologic malignancies. The aim of this article is to provide updated knowledge about bloodstream infections in febrile neutropenia episodes within the Andean region of Latin America.

**Method:**

This retrospective study was based in 6 hospitals in Chile, Ecuador, and Peru and included adult patients with acute leukemia or lymphoma and febrile neutropenia between January 2019 and December 2020.

**Results:**

Of the 416 febrile neutropenia episodes, 38.7% had a bloodstream infection, 86% of which were caused by gram-negative rods, with *Klebsiella pneumoniae*, *Escherichia coli*, and *Pseudomonas aeruginosa* being the most frequently identified bacteria. *K pneumoniae* isolates were more frequently resistant than *E coli* to cefotaxime (65% vs 39.6%), piperacillin-tazobactam (56.7% vs 27.1%), and imipenem (35% vs 2.1%) and were more frequently multidrug resistant (61.7% vs 12.5%). Among *P aeruginosa*, 26.7% were resistant to ceftazidime, piperacillin-tazobactam, and imipenem, and 23.3% were multidrug resistant. Overall 30-day mortality was 19.8%, being higher with vs without a bloodstream infection (26.7% vs 15.3%, *P* = .005). Fever duration was also significantly longer, as well as periods of neutropenia and length of hospital stay for patients with bloodstream infection. Additionally, the 30-day mortality rate was higher for episodes with inappropriate vs appropriate empirical antibiotic therapy (41.2% vs 26.6%, *P* = .139).

**Conclusions:**

Considering the high rates of bacteria-resistant infection and 30-day mortality, it is imperative to establish strategies that reduce the frequency of bloodstream infections, increasing early identification of patients at higher risks of multidrug bacteria resistance, and updating existing empirical antibiotic recommendations.

## Teaser

There are scarce epidemiologic data on febrile neutropenia infections in people with hematologic malignancies in Latin America. This retrospective and multicenter study offers updated knowledge regarding bloodstream infections, predominantly consisting of gram-negative rods with high rates of antibiotic resistance.

Febrile neutropenia (FN) is a medical emergency frequently observed among patients with acute leukemia (AL) and lymphoma (L) after high-intensity myeloablative chemotherapy. It is associated with high morbidity and mortality rates, increasing costs, and the use of health care resources [1–5]. During FN episodes, bloodstream infections (BSIs) have been documented in 30% to 50% of cases, either by gram-negative rods (GNRs) or gram-positive cocci (GPC), with a predominance of one over the other changing over time [6]. Patients with FN episodes require urgent empirical antibiotic therapy, based on international, national, or local guidelines [7, 8]. Patients with AL and L frequently receive antimicrobial prophylaxis, which increases selective pressure on them, leading to antibiotic resistance [9].

Antimicrobial resistance is a global health threat [10]. In particular, GNRs have emerged as an important issue in different clinical settings [11], including hematologic cases [12]. Several resistance mechanisms have been identified, such as extended-spectrum β-lactamases (ESBLs), amp-C β-lactamases, carbapenemases, among others, and very few antimicrobial therapy options are available [13]. Additionally, greater bacterial resistance was accelerated during the COVID-19 pandemic, as were BSIs due to multidrug-resistant (MDR) bacteria at many health care centers, especially in the intensive care units [14, 15].

Considering the aforementioned scenario, it is highly likely that bacterial resistance has increased among patients with AL and L in health care centers within the Andean region, as has been described for other countries in Asia, Europe, and North America and in a few Latin American countries [16–18]. Consequently, some of the empirical antibiotic therapies recommended in current guidelines could no longer be appropriate [19], at least for a proportion of the neutropenic cases, putting patients at increased risks of missing days of adequate treatment with the threat of severe sepsis and death. There is a scarcity of published epidemiologic data from the Andean region of Latin America, and existing reports are out of date [20–22]. Hence, there is an urgency to update corresponding epidemiologic information by center, country, and region.

The aim of this research was to offer updated knowledge about the epidemiology and clinical and microbiological characteristics of BSI in FN episodes observed in patients with AL and L in 6 selected centers within the Andean region.

## METHODS

### Study Design

This retrospective study was conducted at the following 6 reference hospitals in main cities from 3 countries within the Andean region of Latin America:

- Sótero del Río and UC-CHRISTUS in Santiago, Chile
- Eugenio Espejo in Quito and SOLCA in Guayaquil, Ecuador
- Guillermo Almenara and Instituto Nacional de Enfermedades Neoplásicas in Lima, Peru

The study population included adults with AL or L (≥18 years old) who were receiving chemotherapy and experienced FN episodes between 1 January 2019 and 31 December 2020. Patients undergoing hematopoietic cell transplantation (HCT) were excluded.

### Ethical Approvals

Before data collection began, the institutional review board at Pontificia Universidad Católica de Chile, the coordinating center, as well as all local institutional review boards from the participating centers approved the protocol and waived consent for enrollment considering the retrospective design.

### Patient Consent

Our study did not include factors necessitating patient consent.

### Procedures

Based on a survey conducted at the beginning of the study, it was determined that microbiological diagnostic capabilities, clinical approaches, and antibiotic availability for empiric therapy were similar among the 6 centers. However, differences were identified in the strategies of antibiotic-resistant bacteria colonization surveillance and antibiotic prophylaxis use.

The BSI diagnostic approach at each health care center included at least 2 sets of aerobic blood cultures. The susceptibility test was carried out via automated or manual methods. Regarding the automated approach, 3 hospitals used Vitek 2 (bioMérieux) and 1 used Phoenix (BD Diagnostics). With respect to the manual methods, 1 hospital used disks diffusion, and another used agar dilution. All the laboratories used techniques for ESBL detection, as well as for carbapenemases, such as Carba-NP, Blue Carba, and the modified carbapenem inactivation method. Immunochromatography tests were performed to identify carbapenemases.

Documenting clinical sources of infections was based on published definitions [23]. In general, antibiotic management recommendations by FN international guidelines [7] were used as references at each center. The following were recorded and collected via a standardized form and the REDCap platform: demographic and clinical characteristics, main comorbidities, AL/L disease, bacterial colonization, antibiotic prophylaxis, BSI, antibiotic susceptibilities, clinical focus, therapy, length of hospital stay, fever and neutropenia duration, and mortality.

### Definitions

#### Febrile Neutropenia

FN was defined as an oral temperature >38.3 °C or 2 consecutive readings >38.0 °C for 2 hours and an absolute neutrophil count <500 cells/mm^3^ or one expected to fall to <500 cells/mm^3^ during the next 48 hours [7, 24].

#### Bloodstream Infection

BSI was defined as bacteria identification from blood cultures while the patient remained neutropenic in the same FN episode. Contamination was excluded, which was defined as common skin flora species recovered from a single blood culture bottle (eg, coagulase-negative *Staphylococcus*). Early BSI was defined when the event occurred from the first to fourth days of FN and late BSI when it occurred from the fifth day onward.

#### Bacterial Colonization

Bacterial colonization was defined as bacterial identification obtained from rectal swabs before the FN episode.

#### Multidrug Resistance

Multidrug resistance was defined as bacteria resistant to >1 antibiotic in >3 categories [25].

#### Extensive Drug Resistance

Extensive drug resistance was defined if the bacteria was checked for susceptibility to all relevant antibiotics and found nonsusceptible to ≥1 agent in all but <2 categories [25].

#### Inappropriate Antimicrobial Therapy.

Inappropriate antimicrobial therapy involved the absence of antimicrobial prescription to which the microorganism was susceptible or the use of an agent to which the organism was resistant [26].

### Outcomes

The main clinical outcomes were days of fever, neutropenia, length of hospital stay, and 30-day mortality.

### Statistical Analysis

Patient demographics and characteristics of the FN episodes are presented by count and percentage for categorical data and mean and SD or median and range for continuous data.

The chi-square or Fisher exact test was used for dichotomous variables and the *t* test for continuous variables. Statistical analysis was performed with Stata SE 15.0 software for Windows (StataCorp). *P* ≤ .05 was considered statistically significant.

## RESULTS

### General Characteristics

We identified 271 patients with AL or L who experienced an FN episode. Overall, 137 (50.6%) patients were from Ecuador (117 at Eugenio Espejo and 20 at SOLCA), 70 (25.8%) from Peru (38 at Guillermo Almenara and 32 at Instituto Nacional de Enfermedades Neoplásicas), and 64 (23.6%) from Chile (39 at Sótero del Río and 25 at UC-CHRISTUS). Female patients were predominant (141 [52%] vs 130 [48%]), but in Chilean hospitals males were predominant (39 [60.9%] vs 25 [39.1%]). The mean ± SD age was 41.2 ± 17.4 years, and AL was more frequent than L (217 [80.1%] vs 54 [19.9%]). Acute lymphatic leukemia predominated in Eugenio Espejo and Guillermo Almenara, while acute myeloid leukemia was more frequent in the Instituto Nacional de Enfermedades Neoplásicas and UC-CHRISTUS, and non-Hodgkin lymphoma in Sótero del Río and SOLCA. Regarding comorbidities, the median Charlson index was 2 (range, 0–10), being the highest in Sótero del Río at 4.0 (2–10). The most prevalent nonhematologic comorbidities were diabetes mellitus at 18 cases (6.6%), HIV/AIDS at 15 (5.5%), renal failure at 6 (2.2%), and connective tissue disorders at 6 (2.2%).

### FN Episodes

In total, 416 FN episodes were registered. As shown in Table 1, female gender and acute lymphatic leukemia were more frequent. The highest number of episodes was identified in a health center from Ecuador, followed by 1 in Peru and 1 in Chile. Some degree of mucositis was registered in 217 (52.4%) FN episodes, and patients had a central venous catheter in place in 136 (32.8%) episodes. Eighty-eight (21.1%) episodes had a known bacterial colonization: 67 (16.1%) GNR, 14 (3.4%) GPC, and 7 (1.9%) GNR and GPC.

**Table 1.** Characteristics of 416 Febrile Neutropenia Episodes

	No. (%)
Female gender	221 (53.1)
Hematologic malignancy	
Acute lymphatic leukemia	194 (46.6)
Acute myeloid leukemia	147 (35.3)
Non-Hodgkin lymphoma	74 (17.8)
Hodgkin disease	1 (0.2)
Hospital^a^	
EE, Ecuador	197 (47.4)
GA, Peru	65 (15.6)
SR, Chile	52 (12.5)
INEN, Peru	38 (9.1)
UC, Chile	38 (9.1)
SG, Ecuador	26 (6.3)
Mean ± SD	
FN episodes per patient	1.5 ± 0.8
Days between FN and more recent chemotherapy	8.3 ± 7.6
FN diagnosed during hospitalization	232 (55.9)
Absolute neutrophil count at FN diagnosis, cells/mm^3^, median (range)	60 (0–501)
Clinical service where was admitted for FN	
General ward	247 (59.4)
Intensive care units	168 (40.4)
Episodes receiving antibiotic prophylaxis at the time of FN diagnosis	94 (22.6)
Ciprofloxacin or levofloxacin	39 (41.5)
Trimethoprim/sulfamethoxazole	36 (38.3)
Amoxicillin + clavulanic acid	19 (20.2)
Episodes receiving antibiotic nonprophylaxis (for proven/suspected infection) at time of FN diagnosis	58 (13.9)

Abbreviation: FN, febrile neutropenia.

^a^EE, Eugenio Espejo, Quito; GA, Guillermo Almenara, Lima; INEN, Instituto Nacional de Enfermedades Neoplásicas, Lima; SG, SOLCA, Guayaquil; SR, Sótero del Río, Santiago; UC, UC-CHRISTUS, Santiago.

An overall 152 (36.5%) episodes were from patients with antibiotics at the time of FN diagnosis, 94 (22.6%) as prophylaxis, and 58 (13.9%) for proven or suspected infection. Prophylaxis use was different among participating centers: SOLCA, 73.1%; UC-CHRISTUS, 55.3%; Sótero del Río, 53.8%; Eugenio Espejo, 16.8%; Guillermo Almenara, 12.3%; and no prophylaxis at Instituto Nacional de Enfermedades Neoplásicas. Regarding clinical severity of FN episode, 168 (40.4%) episodes required intensive care unit admission upon FN diagnosis.

### Antibiotic Therapy

The most frequently prescribed drug for initial empirical antibiotic therapy was piperacillin-tazobactam in 163 (39.2%) episodes, followed by imipenem or meropenem in 92 (22.1%) and ceftazidime or cefepime in 60 (14.4%). Ciprofloxacin, ampicillin-sulbactam, or other antibiotics were empirically indicated for 101 (24.3%) episodes. Antibiotic monotherapy was prescribed for 140 (33.7%) episodes, while 131 (31.5%) were associated with vancomycin, 51 (12.3%) with metronidazole, 25 (6%) with amikacin, 20 (4.8%) with vancomycin plus amikacin, and other combinations for 49 cases (11.8%). No differences in antibiotic prescription were observed between those who were receiving and not receiving antibiotic prophylaxis.

The mean number of antibiotic prescriptions per FN episode was 2.2 ± 1.1. Empirical antibiotic therapy was prescribed for the 416 episodes and was used for 5.6 ± 3.9 days. The prescription was changed in 274 cases: due to fever in 193 (70.4%) and culture-driven decisions in 81 (29.3%). After 7.1 ± 6.6 days, an adjustment of antibiotic prescription was necessary for 174 episodes, where 89 (60.5%) were related to fever and 55 (37.4%) to culture results. Then, after 6.7 ± 4.9 days, antibiotic therapy was changed in 58 episodes, where 33 (56.9%) were related to persistent fever. Finally, after 7.3 ± 3.5 days, another change had to be made in 17 episodes, where 10 (58.8%) cases were related to continuing febrile episodes.

### Microbiological Identification

Regarding FN etiology, the cause of fever was identified in 330 (79.3%) episodes. A BSI was documented in 161 cases (38.7%), while a focus of infection was registered without a documented BSI in 152 episodes (36.5%). A bacterial non-BSI, viral, and/or fungal infection was identified in 17 (4.1%) episodes, and fever was of unknown origin in 86 (20.7%) cases.

Regarding the 161 BSIs, 99 (61.5%) were early BSIs and 62 (38.5%) were late. A lower frequency of early BSI was detected among the 94 episodes that were under antibiotic prophylaxis, as compared with those diagnosed among the 322 episodes without antibiotic prophylaxis (16 [18%] vs 83 [25.8%], *P* = .12). Additionally, GNRs and GPCs were less frequently identified in early BSI among those receiving prophylaxis vs those who were not (15 [16%] vs 69 [21.4%], *P* = .2; 1 [1.1%] vs 14 [4.3%], *P* = .2, respectively).

A total of 198 bacteria were isolated from blood cultures, of which 108 (54.5%) were identified in early BSI and 90 (45.4%) in late BSI. As shown in Table 2, GNRs were more frequently identified than GPCs. *Klebsiella pneumoniae*, *Escherichia coli*, and *Pseudomonas aeruginosa* were the most frequent GNRs, while *Staphylococcus epidermidis*, *Staphylococcus aureus*, and *Enterococcus faecium* were the most frequent GPCs.

**Table 2.** Frequency of Bacteria Identified in BSI: Total and Separated by Early and Late BSI

	Total	Early BSI (1–4 d)	Late BSI (≥5 d)
GNR	172 (86.0)	93 (84.5)	79 (87.8)
Enterobacterales	129 (75.0)	73 (78.5)	56 (70.9)
*Klebsiella pneumoniae*	60 (46.5)	34 (46.6)	26 (46.4)
*Escherichia coli*	48 (37.2)	27 (37.0)	21 (37.5)
*Enterobacter cloacae*	5 (3.9)	3 (4.1)	2 (3.6)
*Enterobacter aerogenes*	3 (2.3)	2 (2.7)	1 (1.8)
*Serratia marcescens*	3 (2.3)	…	3 (5.4)
*Aeromonas hydrophila/caviae*	2 (1.6)	2 (2.7)	…
*Klebsiella oxytoca*	2 (1.6)	2 (2.7)	…
*Proteus vulgaris*	1 (0.8)	…	1 (1.8)
*Citrobacter freundii*	1 (0.8)	…	1 (1.8)
*Leminorella richardii*	1 (0.8)	…	1 (1.8)
*Aeromonas* sp	1 (0.8)	1 (1.4)	…
*Enterobacter* sp	1 (0.8)	1 (1.4)	…
*Proteus miralbilis*	1 (0.8)	1 (1.4)	…
Nonfermenting	43 (25.0)	20 (21.5)	23 (29.1)
*Pseudomonas aeruginosa*	30 (69.8)	16 (80.0)	14 (60.9)
*Acinetobacter baumannii*	3 (7.0)	…	3 (13.0)
*Acinetobacter lwoffii*	3 (7.0)	…	3 (13.0)
*Burkholderia cepacia complex*	2 (4.7)	…	2 (8.7)
*Stenotrophomonas maltophilia*	2 (4.7)	1 (5.0)	1 (4.3)
*Pseudomonas putida*	1 (2.3)	1 (5.0)	…
*Sphingomonas paucimobilis*	1 (2.3)	1 (5.0)	…
*Acinetobacter haemolyticus*	1 (2.3)	1 (5.0)	…
GPC^a^	26 (14.0)	15 (15.5)	11 (12.2)
*Staphylococcus epidermidis*	12 (46.4)	7 (46.7)	5 (45.5)
*Staphylococcus aureus*	7 (25.0)	6 (40)	1 (9.1)
*Enterococcus faecium*	4 (14.3)	1 (6.7)	3 (27.3)
*Staphylococcus haemolyticus*	2 (7.1)	…	2 (18.2)
*Streptococcus* group *viridans*	1 (3.6)	1 (6.7)	…

Abbreviation: BSI, bloodstream infection; GNR, gram-negative rod; GPC, gram-positive cocci.

^a^One *S epidermidi*s and 1 *S haemolyticu*s were not included because they were considered contamination.

**Figure 1.**
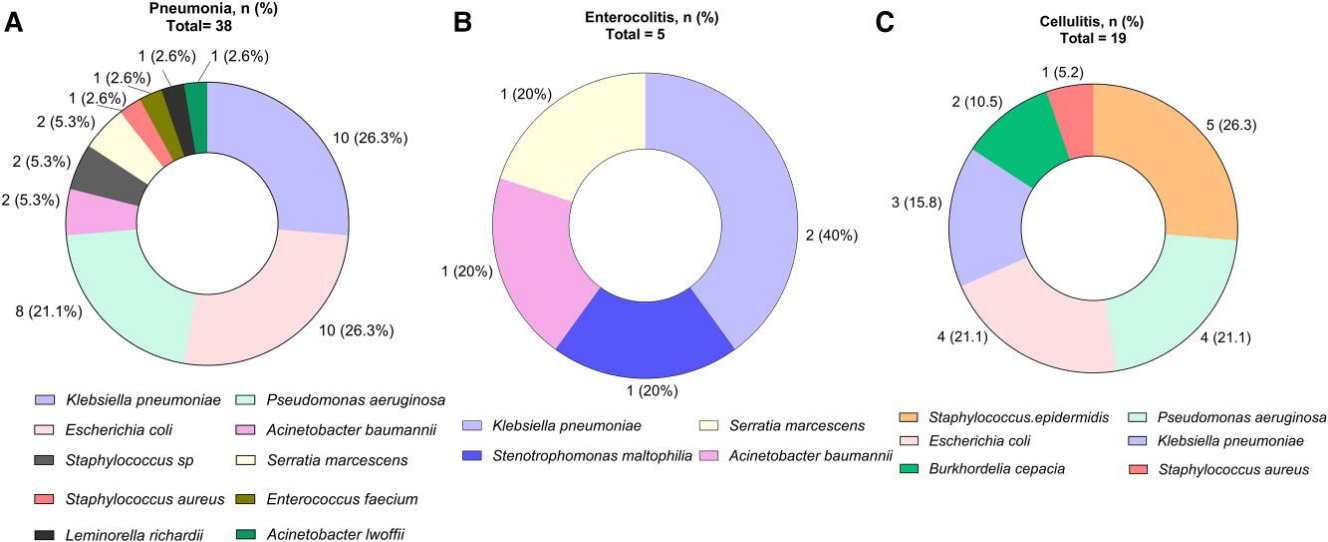
*A–C*, Frequency of bacteria identified in BSI in 38 pneumonia, 5 enterocolitis, and 19 cellulitis cases. BSI, bloodstream infection.

Regarding the 269 FN episodes with a source of infection, 236 (87.7%) were identified during the first 4 days, 33 (12.3%) on day 5 onward, and 117 (43.4%) had a documented BSI. The most frequent was respiratory in 108 (40.1%) episodes, including 94 (34.9%) pneumonia cases. The second was gastrointestinal in 99 (36.8%) cases, including 14 (5.2%) neutropenic enterocolitis cases. The third in frequency was skin and soft tissue in 69 (25.6%), including 36 (13.4%) cellulitis cases. Figure 1 shows the bacteria identified in the BSIs of pneumonia, enterocolitis, and cellulitis episodes.

### Antibiotic Resistance

With regard to the 172 GNRs identified in BSI, 72 (41.9%) were resistant to cefotaxime, 58 (33.7%) to piperacillin-tazobactam, 55 (32%) to cefepime, and 39 (22.7%) to carbapenems. In relation to their resistance mechanism, ESBLs were reported in 30 (17.2%), carbapenemases in 19 (11%)—16 *K pneumoniae* carbapenemase (KPC), 2 New Delhi metallo-beta-lactamase (NDM), 1 Verona integron-mediated-beta-lactamase (VIM)—AmpC in 3 (1.7%), and permeability alterations in 1 (0.6%).

Specifically, of the 129 Enterobacterales, 56 (43.4%) were resistant to cefepime, 50 (38.8%) to cefotaxime, 47 (36.4%) to piperacillin-tazobactam, and 28 (21.7%) to carbapenems. In relation to the resistance mechanism, ESBL was reported in 19 (14.7%) cases, carbapenemases in 14 (10.8%), and AmpC in 3 (2.3%). *K pneumoniae* had the highest antibiotic resistance rate, with 75% resistance to ampicillin-sulbactam, 65% to cefotaxime, 60% ciprofloxacin, 56.7% cefepime and piperacillin-tazobactam, 43.3% meropenem, and 35% imipenem. *E coli* showed 56.3% resistance to ampicillin-sulbactam, 39.6% to cefotaxime, 33.3% cefepime, 27.1% piperacillin-tazobactam, 22.9% ciprofloxacin, and 2.1% to imipenem and meropenem.

*P aeruginosa* showed 30% resistance to ciprofloxacin and 26.7% to ceftazidime, cefepime, piperacillin-tazobactam, imipenem, and meropenem; this includes the identification of 2 (6.7%) permeability alterations, 1 (3.3%) carbapenemase, and 1 (3.3%) ESBL.

**Figure 2.**
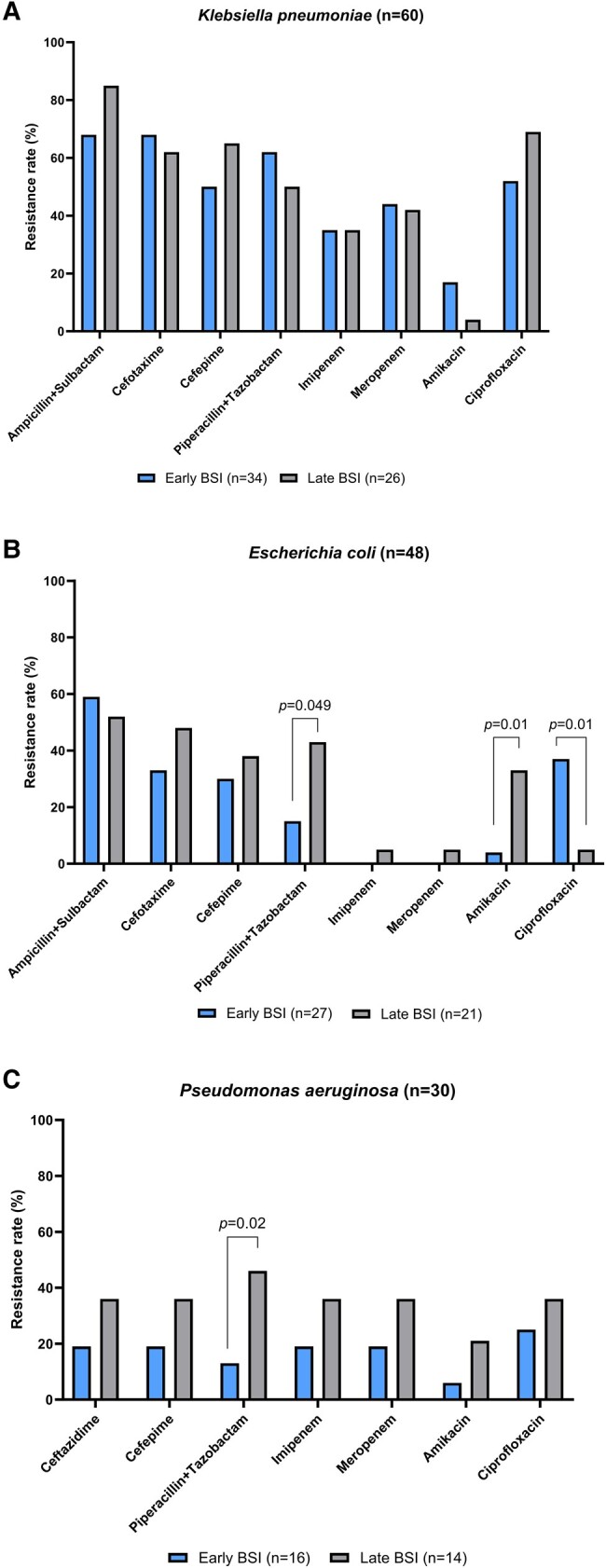
*A–C*, Frequency of antibiotic resistance for *Klebsiella pneumoniae*, *Escherichia coli*, and *Pseudomonas aeruginosa* identified in BSI, comparing early BSI (1–4 days) vs late (>4 days) during episodes of febrile neutropenia. BSI, bloodstream infection.

Figure 2 compares resistance rates of *K pneumoniae*, *E coli*, and *P aeruginosa* isolated in early vs late BSI, with a significant increase in *E coli* resistance to piperacillin-tazobactam and amikacin, as well as *P aeruginosa* resistance to piperacillin-tazobactam. A significant decrease in *E coli* ciprofloxacin resistance was also observed.

Based on antibiotic resistance definitions [25] for the 60 *K pneumoniae*, 48 *E coli*, and 30 *P aeruginosa*, 37 (61.7%), 6 (12.5%), and 7 (23.3%) were considered MDR, whereas 20 (33.3%), 1 (2%), and 4 (13.3%) were extensively drug resistant. With respect to antibiotic resistance among those who were undergoing antibiotic prophylaxis vs nonprophylaxis, it was higher among *K pneumoniae* for cefotaxime (100% vs 73.9%, *P* = .3), piperacillin-tazobactam (85.7% vs 56.5%, *P* = .2), and imipenem (57.1% vs 34.8%, *P* = .4). However, *E coli* had higher resistance only to cefotaxime (50% vs 33%, *P* = 1), not to the other antibiotics, while *P aeruginosa* did not show any differences in resistance.

Among the 18 episodes colonized with carbapenem-resistant Enterobacterales (CRE), 3 (16.7%) BSIs were detected: 2 KPC and 1 NDM. Moreover, among 70 episodes without CRE colonization, 2 (2.9%) BSIs with KPC were detected.

In relation to the 26 GPCs, 9 of 13 (69.2%) *S epidermidis* were methicillin resistant, and 2 of 4 (50%) *E faecium* were vancomycin resistant. On the contrary, all the *S aureus* were methicillin susceptible.

### Outcomes

Clinical outcomes are presented in Table 3, showing statistically significant unfavorable results for BSI vs non-BSI episodes. In addition, it shows clinical outcomes in terms of the appropriateness of empirical antibiotic therapy for early BSI. Results were unfavorable for episodes involving inappropriate antimicrobial therapy, although without statistically significant differences.

**Table 3.** Clinical Outcomes of Febrile Neutropenia Episodes

	Febrile Neutropenia Episodes				Early BSI (n = 98^a^)			Early GNR BSI (n = 83^a^)		
	Total (n = 416)	BSI (n = 161)	Non-BSI (n = 255)	*P* Value	AAT (n = 64)	IAT (n = 34)	*P* Value	AAT (n = 52)	IAT (n = 31)	*P* Value
Median (range)										
Days of fever	3 (1–44)	5 (1–44)	3 (1–28)	**<.001**	5 (1–24)	5 (1–26)	.053	4 (1–24)	5 (1–26)	.819
Days of neutropenia	8 (1–79)	8.5 (1–79)	8 (1–41)	**.014**	8 (1–60)	8 (1–79)	.988	8.5 (1–60)	8 (1–79)	.985
Length of stay, d	21 (1–122)	25 (1–90)	17 (1–122)	**<.001**	20 (2–90)	19 (1–79)	.794	18 (2–90)	18.5 (1–79)	.442
30-d mortality, No. (%)	82 (19.7)	43 (26.7)	39 (15.3)	**.005**	17 (26.6)	14 (41.2)	.139	17 (32.7)	14 (45.2)	.339

Comparison includes those with and without BSI, early BSI episodes, and early BSI episodes with GNR identification by AAT and IAT. Bold indicates *P* ≤ .05.

Abbreviations: AAT, appropriate antibiotic therapy; BSI, bloodstream infection; GNR, gram-negative rod; IAT, inappropriate antibiotic therapy.

^a^The episode with *Sphingomonas paucimobilis* was excluded from analysis because the antibiogram was not available.

We did not observe differences in the outcomes among those episodes receiving vs not receiving antibiotic prophylaxis.

Finally, a bivariate analysis comparing 61 patients with AL and L who died vs 210 who survived showed that BSI (57.4% vs 38.1%, *P* = .01), GNR BSI (100% vs 77.6%, *P* = .002), and transfers to the intensive care unit (75.6% vs 12.7%, *P* < .01) were significantly associated with 30-day mortality.

## DISCUSSION

This retrospective study revealed a predominance of GNR BSI in FN episodes among patients with AL and L from 6 reference hospitals in Chile, Ecuador, and Peru, with an overall high frequency of antibiotic resistance and high mortality rates.

Results indicated that 38.9% of FN episodes exhibited BSI. This is within the range described for hematologic cases and >14% reported by Parodi et al, according to a multicentric prospective study in Argentina focusing on FN in patients with hematologic and solid organ tumors [27]. This can be explained by the characteristics of the patients in the current study, among which AL was the most frequent hematologic disease, given that its FN episodes are known to be high-risk events typically leading to complications.

The finding of the present study regarding GNR predominance has been reported in several studies from different health care centers but with different magnitudes [27–29], including catheter-related BSI [30]. Higher GNR prevalence indicates a shift with respect to a Chilean study that described a GPC predominance [20]. Some health care centers continue to report a predominance of GPC [28, 31, 32] or an equal distribution of GNR and GPR [33].

Among GNR BSI cases, the present study identified *K pneumoniae*, *E coli*, and *P aeruginosa* as the bacteria with the highest resistance rates to the antibiotics recommended by existing international guidelines [7]. In fact, the resistance rates revealed here were higher than those reported by Cattaneo et al in an Italian multicenter prospective study of patients with AL, which showed incidences of 23.2% for ESBL and 9% for CRE, as well as 21% for multiresistant *P aeruginosa* [34]. Similarly, in a multicenter study in the United States, including FN after chemotherapy and patients with HCT, Zimmer et al identified *E coli*, *K pneumoniae*, and *P aeruginosa* as the most frequent GNRs, with >85% Enterobacterales susceptibility to cefepime, piperacillin-tazobactam, and carbapenems and >90% *Pseudomonas* susceptibility to cefepime and piperacillin-tazobactam, as well as 86% susceptibility to carbapenems [33]. Regarding MDR bacteria, in a retrospective study in a single center from Brazil that included chemotherapy and patients with HCT, the incidence of CRE was 16.6% [32]. Conversely, a multicenter European study noted a CRE incidence of 8.4% [35]. Furthermore, in a retrospective study from 1 health care center in China, Wang et al reported 89 BSIs in 348 FN episodes in patients who received HCT, with a higher frequency of GNRs and an overall 30.1% incidence of MDR and 12.3% of CRE. The latter study demonstrated that patients with high-risk diseases, prolonged neutropenia, and carbapenem-resistant GNRs presented independent risk factors for BSI-related mortality [36]. All these data underscore the importance of better comprehending local epidemiologic data.

In view of the high mortality rates found in the present study—26.7% in BSI episodes and 45.2% in those with GNR BSI who received inappropriate antimicrobial therapy—it is crucial to apply different interventions to decrease BSI rates: (1) use of antibiotic prophylaxis, (2) prompt identification of higher-risk FN episodes for GNR BSI to adapt to our empirical approach, and (3) updated recommendations to timely and effectively treat antibiotic-resistant GNRs when confirmed.

Although this was not a study to evaluate the effectiveness of antibiotic prophylaxis, a tendency to reduce early BSI was observed according to our data. However, given the higher frequency of antibiotic-resistant *K pneumoniae* detected, this practice must be directly evaluated to better identify its advantages and risks. Publications on this topic raise the question regarding the benefits for the ecology of resistance in each center [37]. In our opinion, it is mandatory to implement a program of antibiotic resistance surveillance in relation to prophylaxis use.

Regarding how to identify patients at higher risk for MDR infections, Herrera et al reported a clinical score to stratify risk for BSIs with CRE in patients with cancer and HCT. The scoring system incorporated key risk factors, each of which was assigned a corresponding point value, facilitating a comprehensive risk assessment: >10 days of hospitalization (2 points), prior treatment with antibiotics for >7 days (2 points), and current colonization with KPC carbapenem–producing Enterobacterales (5 points). Patients scoring ≥7 points demonstrated a remarkable specificity of 98.3%, a positive predictive value of 77.7%, and a negative predictive value of 90.9% for CRE bacteremia, signifying a substantial risk. In the absence of any of these factors, the probability of CRE was only 1.9% [38]. For the design of our study, we could not confirm the impact of these factors, but we observed a very low frequency of KPC BSI among those not colonized. Based on our results and considering >20% CRE and *P aeruginosa*, it is strongly recommended to enhance MDR GNR screenings with rectal swabs [39, 40] and customize the optimal therapy against colonizing GNR in local protocols [41]. In a similar way, Garcia-Vidal et al assessed the risk of MDR GNR infection at FN onset in hematologic cases using machine learning at a health care center in Spain and identified other factors: age >45 years, prior antibiotic use, first-ever FN in a hospitalization period, previous admissions for FN, at least 15 prior hospital visits, high-risk hematologic conditions, and hospitalization in a room formerly occupied by patients with MDR GNR isolation [42].

With regard to updating empirical therapy, considerations such as ceftazidime-avibactam, accessible in all 3 countries of the study, could be suggested as empirical treatment for FN in patients colonized by KPC- or OXA-48–producing Enterobacterales [39, 43]. Nonetheless, the clinical outcomes of this approach require future evaluation. Moreover, ceftolozane/tazobactam could be considered an alternative due to its potent activity against *P aeruginosa* strains according to Latin American data [44], and its empirical application in FN has yielded safety data and improved clinical outcomes when compared with cefepime, piperacillin/tazobactam, and meropenem [45].

When interventions are being planned to improve care for patients with FN, epidemiologic data must be available to guide the empirical antibiotic approach. However, caution must be taken to avoid unnecessary broad-spectrum antibiotic prescriptions by using discriminatory tools to decide on de-escalation algorithms upon BSI identification. In a recent study from Peru, implementation of a rapid polymerase chain reaction–based blood culture identification panel had an impact on antibiotic use in this patient setting [46].

The study reported here has some limitations, such as the retrospective design, the different sample sizes at different centers, the differences in the use of antimicrobial prophylaxis, and dissimilar colonization surveillance strategies. Another issue is that some resistance rates were calculated in <30 determinations, which makes it is necessary to evaluate these data with precaution. Finally, during the COVID-19 pandemic, during which this study was being carried out, hospitals were in demand with high occupancy rates, which could have affected patient care. Despite that, the results presented are an alert for physicians in charge of hematologic cases within the region, given that several health care centers could be experiencing similar realities.

In conclusion, it is mandatory to continuously monitor BSIs in FN episodes to better understand the real epidemiology at hand, including, for instance, the finding here regarding the high prevalence of GNR and MDR BSIs in patients with AL and L within the Andean region. It is important to review and adapt existing empirical antibiotic recommendations toward avoiding inappropriate antimicrobial therapy in these vulnerable patients.

## Notes

***Author contributions*.** R. R.: conceptualization, data curation, formal analysis, funding acquisition, investigation, methodology, project administration, resources, supervision, validation, writing–original draft, writing–review and editing. G. S.: conceptualization, investigation, validation, writing–review and editing. G. P.-L.: conceptualization, investigation, validation, writing–review and editing. M. P. I.: conceptualization, investigation, validation, writing–review and editing. D. P.: conceptualization, investigation, validation, writing–review and editing. C. S.-S.: conceptualization, investigation, validation, writing–review and editing. M. J. O.: conceptualization, design of the case report form for REDCap, and statistical analysis. J. F.: data curation and supervision data episodes of centers participants. M. G.: investigation and data curation. R. S.-A.: investigation and data curation. P. C. A.: investigation and data curation. L. D.: investigation and data curation. F. A. R.: investigation and data curation. P. G.: conceptualization, investigation, validation, writing–review and editing.

***Financial support*.** This work was supported by Pfizer (grant 68727191).
